# Proton‐Switching Regulates Interfacial Iodine Chemistry for Long‐Life Zinc–Iodine Batteries

**DOI:** 10.1002/anie.4465475

**Published:** 2026-08-05

**Authors:** Feifei Wang, Guoqin Liu, Yuhang Zhuang, Peng Zhang, Zhengwei Li, Songshan Bi, Ahmad Bagheri, Angelika Wrzesińska‐Lashkova, Neda Kargarfard, Peyman Rostami, Yana Vaynzof, Bernd Plietker, Mingchao Wang, Ali Shaygan Nia, Xinliang Feng

**Affiliations:** ^1^ Max Planck Institute For Microstructure Physics Halle (Saale) Germany; ^2^ Center for Advancing Electronics Dresden (cfaed) and Faculty of Chemistry and Food Chemistry Technische Universität Dresden Dresden Germany; ^3^ Chair of Organic Chemistry I Faculty of Chemistry and Food Chemistry Technische Universität Dresden Dresden Germany; ^4^ Chair for Emerging Electronic Technologies Technische Universität Dresden Dresden Germany; ^5^ Leibniz Institute for Solid State and Materials Research Dresden Dresden Germany; ^6^ Leibniz Institute of Polymer Research Dresden (IPF) Institute of Physical Chemistry and Polymer Physics Dresden Germany; ^7^ State Key Laboratory of Advanced Waterproof Materials & Guangdong Provincial Key Laboratory of Nano‐Micro Materials Research School of Advanced Materials Peking University Shenzhen Graduate School Shenzhen China

**Keywords:** electrochemistry, energy storage, zinc‐iodine batteries, shuttle effect, protonation

## Abstract

Aqueous zinc–iodine (Zn–I_2_) batteries are promising candidates for safe and cost‐effective energy storage, yet their practical application is limited by uncontrolled iodine speciation at electrified interfaces, driving severe polyiodide shuttling and parasitic reactions that cause rapid capacity fading. Existing strategies predominantly rely on static confinement or adsorption, which cannot adapt to evolving iodine speciation during charge–discharge, causing a trade‐off between shuttle suppression and redox kinetics. In this study, we report a proton‐switching strategy that regulates interfacial iodine chemistry via electrochemically driven protonation–deprotonation within an imine‐linked two‐dimensional polymer framework, thereby dynamically rewiring interfacial electrostatics during cycling. During discharge, protonation of imine generates positively polarized C═NH^+^ sites that stabilize I^−^ through electrostatic interactions, enabling controlled reduction of polyiodides without accumulation. Upon charging, deprotonation restores the neutral framework, favoring polyiodide stabilization and efficient iodine oxidation. As a result, the constructed Zn–I_2_ battery delivers 51 000 cycles at 20 A g^−1^ at 25°C and sustains over 70 000 cycles at −20°C. This durability is retained at a high iodine loading of 35.7 mg cm^−2^, delivering an areal capacity of 5 mAh cm^−2^ over 2000 cycles with negligible decay, placing this system among the most durable Zn–I_2_ batteries.

## Graphical abstract

A proton‐switchable imine‐linked framework is developed to dynamically regulate interfacial iodine chemistry in aqueous Zn–I_2_ batteries, where reversible protonation/deprotonation switches the host affinity between iodide and polyiodide species. This state‐dependent regulation suppresses shuttle‐induced side reactions while preserving fast iodine redox kinetics, enabling ultralong cycling, high self‐discharge resistance, and practical high‐loading pouch‐cell operation.

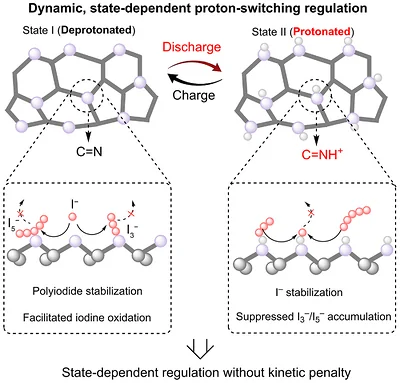

## 1 Introduction

Zinc–iodine (Zn–I_2_) batteries are governed by a reversible solution‐phase redox equilibrium between I_2_ and polyiodide species (I_3_^−^/I_5_^−^), establishing a solution‐mediated electrochemical pathway distinct from conventional solid‐state conversion systems [1, 2, 3, 4, 5, 6]. The intrinsic solubility and dynamic interconversion of iodide (I^−^) ions enable rapid electron transfer and high‐rate performance, yet the same mobility also destabilizes the system as polyiodide species diffuse away from the electrode interface and disperse throughout the electrolyte. The shuttling of polyiodide species between the cathode and the Zn anode triggers parasitic interfacial reactions, leading to the loss of active iodine and progressive capacity fading [7]. This intrinsic conflict between fast redox kinetics and uncontrolled polyiodide migration remains the central challenge of Zn–I_2_ batteries.

To mitigate the shuttle effect, extensive efforts have focused on immobilizing polyiodides via porous confinement or adsorption using carbon hosts [8, 9, 10], and polar frameworks [11, 12, 13, 14, 15, 16], as well as on accelerating iodine redox conversion using molecular electrocatalysts [17, 18]. Despite material diversity, these strategies fundamentally rely on static, state‐independent interactions that attempt to confine iodine species at the interface. While stronger binding can suppress dissolution, it often over‐stabilizes intermediates and hinders redox reversibility. Conversely, weaker interactions fail to effectively prevent polyiodide migration (Scheme 1a) [19]. Therefore, a fundamental trade‐off persists between immobilization strength and redox kinetics. More importantly, these approaches treat polyiodides as undesirable species to be suppressed, rather than dynamic intermediates within the I^−^/I_2_/polyiodides equilibrium. This static design paradigm overlooks the inherently dynamic nature of iodine chemistry, in which speciation continuously evolves under electrochemical conditions.

**SCHEME 1:**
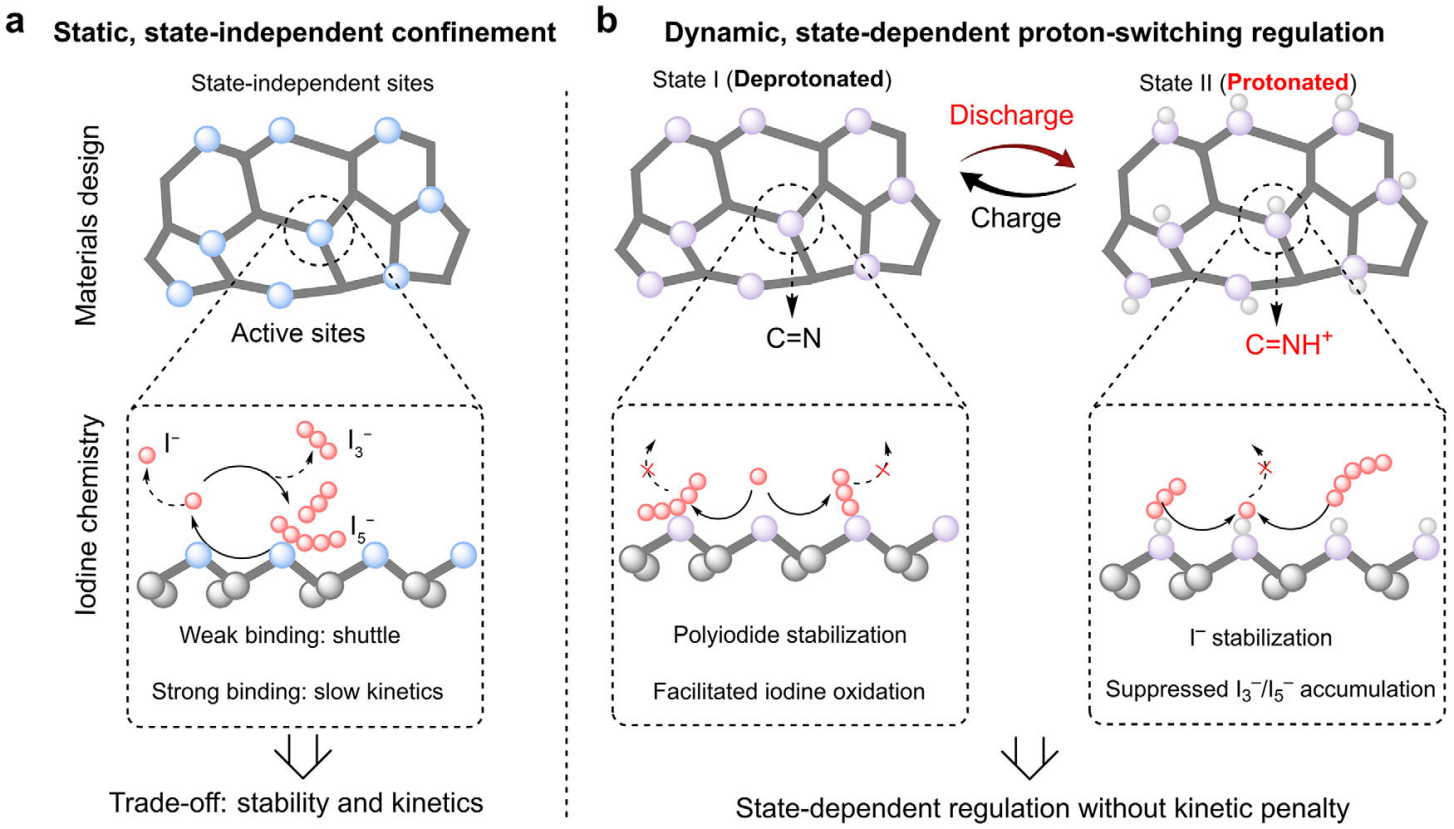
Conceptual illustration of proton‐switch‐regulated iodine chemistry in Zn–I_2_ batteries. (a) Conventional static confinement relies on state‐independent adsorption, resulting in a trade‐off between polyiodide suppression and redox kinetics. (b) Proton‐switching mechanism in the imine‐linked framework, where reversible protonation and deprotonation dynamically modulate interfacial electrostatics, switching the adsorption preference between I^−^ and polyiodide species during discharge and charge, respectively, enabling state‐dependent iodine regulation without kinetic penalty.

Here, we introduce a proton‐switching strategy that enables state‐dependent iodine chemistry at the electrified interface. This mechanism is realized using an imine‐linked two‐dimensional (2D) polymer (ImPh) as a proton‐responsive interfacial framework, whose crystalline structure provides periodically arranged and electrochemically stable proton‐responsive sites for regulating interfacial iodine–species binding (Scheme 1b). During discharge, protonation generates positively polarized C═NH^+^ sites that stabilize I^−^ species by electrostatic interaction and suppress polyiodide accumulation. Upon charging, deprotonation restores the neutral conjugated framework, promoting controlled polyiodide formation and efficient iodine oxidation. In this way, the ImPh framework introduces a dynamic, state‐dependent regulation mechanism that adapts to the evolving iodine chemistry during cycling, thereby overcoming the trade‐off between strong immobilization and redox reversibility. As a result, the constructed Zn–I_2_ cells based on ImPh enable ultralong cycling stability of 51 000 cycles at 20 A g^−1^ at 25°C and sustain over 70 000 cycles at −20°C. Even under practical conditions, a high iodine loading of 35.7 mg cm^−2^ delivers an areal capacity of 5 mAh cm^−2^ and sustains stable cycling over 2000 cycles with negligible decay, surpassing the most durable aqueous Zn–I_2_ configurations. These results establish proton‐switch regulation as an effective interfacial strategy for controlling soluble iodine intermediates beyond static adsorption.

## 2 Results and Discussion

ImPh was synthesized through solvothermal Schiff‐base condensation of a tritopic aldehyde and p‐phenylenediamine, yielding a crystalline imine‐linked 2D polymer framework. The structural characteristics of ImPh were first examined to clarify its potential for regulating iodine chemistry. The ImPh features an imine‐linked porous framework with periodically arranged C═N units along the pore walls, providing both confined channels (∼3.2 nm) for iodine accommodation and chemically accessible adsorption sites (Figure 1a). The crystallinity of the framework is confirmed by powder x‐ray diffraction (PXRD), which agrees well with the simulated structure (Figure S1a). Nitrogen adsorption–desorption measurements further reveal a porous structure with a Brunauer–Emmett–Teller (BET) surface area of 47.2 m^2^ g^−1^ (Figure S1b), indicating the presence of accessible internal channels for guest molecules. Scanning electron microscopy (SEM) images show that ImPh forms aggregated particles with rough surfaces (Figure S1c), while energy‐dispersive x‐ray spectroscopy (EDS) mapping confirms the uniform distribution of nitrogen and carbon within the framework (Figure S1d). These results establish ImPh as a chemically active framework for iodine species.

**FIGURE 1:**
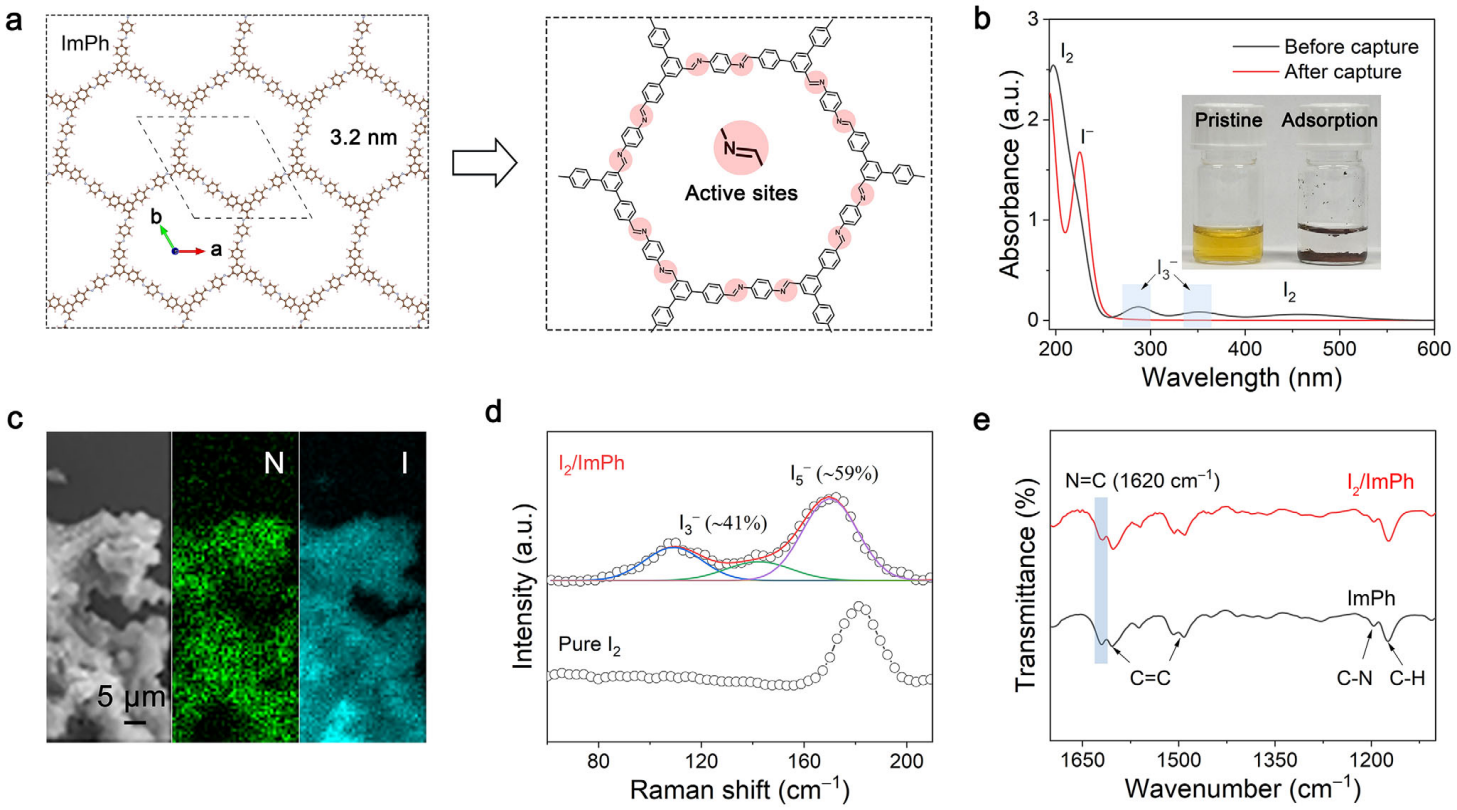
Structural features and iodine‐species interactions of ImPh. (a) Structural model of ImPh and the pore environment with imine active sites. (b) UV–vis absorption spectra of I_2_/H_2_O solution before and after contact with ImPh, with photographs of the corresponding solutions. (c) SEM image and EDS elemental mapping of ImPh after iodine adsorption. (d) Raman spectra of pure I_2_ and I_2_/ImPh. (e) FTIR spectra of pristine ImPh and I_2_/ImPh.

The iodine uptake behavior of ImPh was then examined in aqueous solution. Upon contact with iodine, the characteristic yellow color of the solution gradually faded, accompanied by the formation of a dark solid phase, indicating rapid uptake of iodine species (Figure 1b, inset). The pristine solution exhibits characteristic absorption bands of molecular iodine and polyiodide species in ultraviolet–visible (UV–vis) spectroscopy, whereas these signals are largely suppressed after treatment with ImPh (Figure 1b), indicating efficient removal of soluble iodine species from the electrolyte. A similar attenuation is observed for pre‐formed I_3_^−^ solutions (Figure S2), confirming effective capture of dissolved polyiodide species.

The preserved particle morphology after iodine immobilization, together with the homogeneous iodine distribution revealed by EDS mapping, indicates that iodine species are uniformly incorporated within the ImPh framework rather than deposited on the surface (Figure 1c). The Raman spectra reveal a redistribution of iodine speciation upon interaction with ImPh. Pristine iodine exhibits the characteristic vibration of molecular I_2_, whereas the I_2_/ImPh composite shows a weakened and slightly shifted I_2_ band together with the emergence of additional bands at ∼110 and ∼170 cm^−1^, corresponding to I_3_^−^ and I_5_^−^ species, respectively (Figure 1d) [20]. Deconvolution analysis indicates a dominant I_5_^−^ contribution (∼59%), suggesting that ImPh preferentially stabilizes higher‐order polyiodide species rather than merely physically adsorbing molecular iodine.

Attenuated total reflectance Fourier‐transform infrared (ATR‐FTIR) spectroscopy further confirms the structural integrity of the framework. The ImPh sample exhibits a characteristic C═N stretching vibration at ∼1620 cm^−1^, confirming the presence of imine linkages within the framework. After iodine uptake, this feature remains clearly identifiable, while the overall spectral profile is largely preserved (Figure 1e), indicating that the framework structure is maintained. Consistently, the powder XRD pattern of the I_2_/ImPh composite shows the retained framework diffraction feature after iodine loading (Figure S3), further supporting the structural robustness of ImPh during iodine incorporation.

To elucidate the origin of the observed iodine uptake and speciation behavior, density functional theory (DFT) calculations were performed to compare the adsorption energy of iodine species on ImPh and protonated ImPh (HImPh) (Figure 2a). On ImPh, I^−^ adsorption is energetically unfavorable, with a positive adsorption energy of 0.75 eV, whereas I_2_, I_3_^−^, and I_5_^−^ are all stabilized, with adsorption energies of −0.24, −0.44, and −0.47 eV, respectively. This result indicates that neutral ImPh preferentially accommodates molecular iodine and polyiodide species rather than I^−^, likely owing to the greater charge delocalization and higher polarizability of polyiodides, which enable stronger electronic interactions with the conjugated imine framework than I^−^. Upon protonation, the adsorption preference is reversed. On protonated HImPh, I^−^ becomes the most strongly stabilized species, with an adsorption energy of −0.87 eV, while the adsorption of I_3_^−^ and I_5_^−^ is weakened to −0.19 and −0.03 eV, respectively. Although I_2_ also shows enhanced adsorption on HImPh, the key trend relevant to the discharge process is the marked stabilization of I^−^, accompanied by weakened binding of polyiodide intermediates. This protonation‐dependent redistribution of adsorption energies supports a switch from polyiodide stabilization on neutral ImPh to I^−^ stabilization on HImPh, consistent with regulated polyiodide‐to‐iodide conversion during discharge.

**FIGURE 2:**
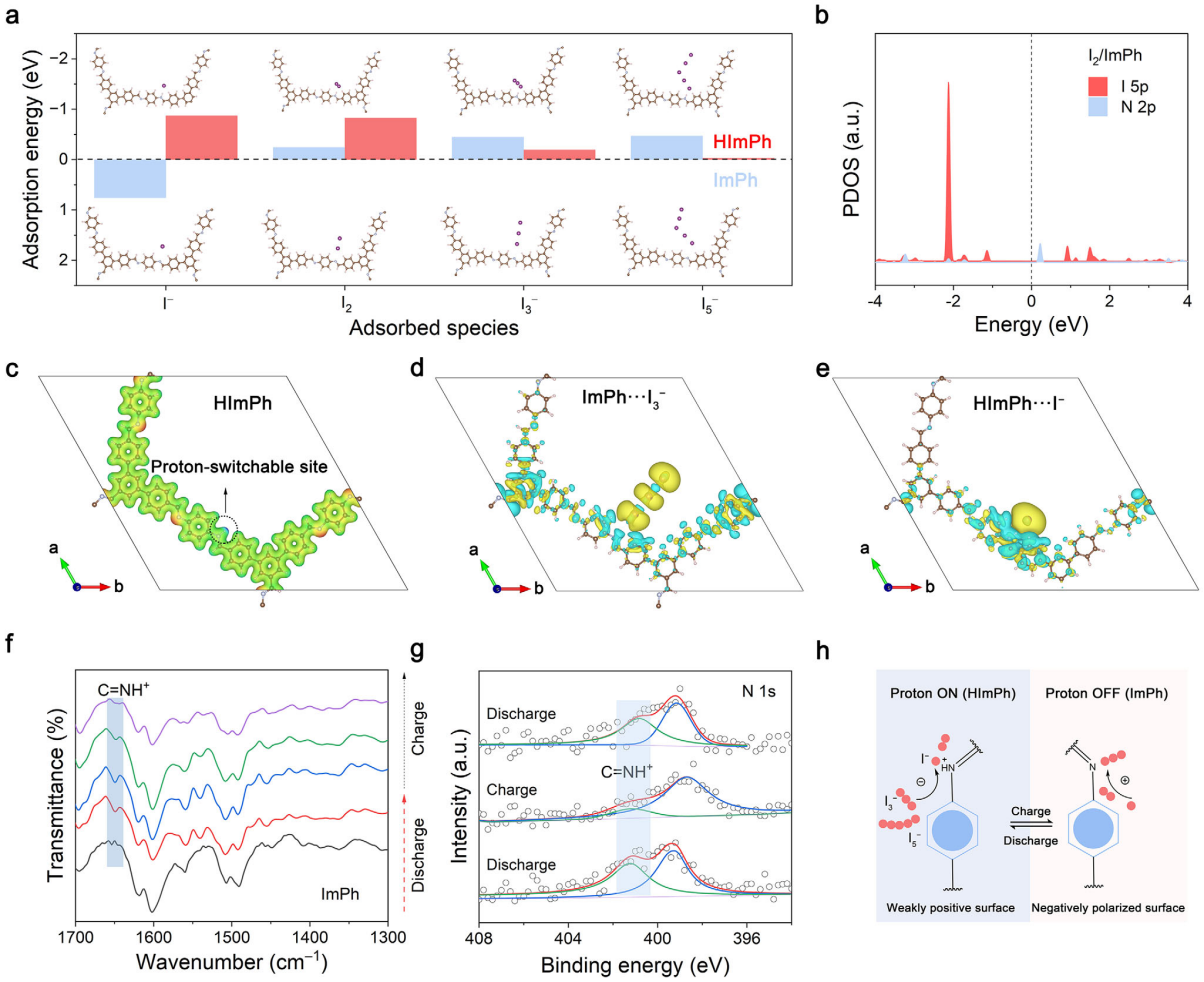
Proton‐switching regulation of iodine‐species binding on ImPh. (a) Calculated adsorption energies of I^−^, I_2_, I_3_^−^, and I_5_^−^ on deprotonated ImPh and protonated HImPh. (b) PDOS of I 5p and N 1s orbitals in the I_2_/ImPh system. (c) Charge density distribution of protonated HImPh. (d) Charge density difference for I_3_^−^ adsorbed on ImPh. (e) Charge density difference for I^−^ adsorbed on HImPh (yellow and cyan regions correspond to electron accumulation and depletionrespectively). (f) Ex situ ATR‐FTIR spectra in the C═N region of ImPh under different electrochemical states. (g) N 1s XPS spectra of ImPh electrodes at charged and discharged states. (h) Schematic illustration of proton‐switching regulation of iodine adsorption on ImPh.

The electronic origin of the iodine–framework interaction was further examined by density of states (DOS) analysis. The partial density of states (PDOS) shows that iodine adsorption introduces pronounced electronic coupling with the imine nitrogen, as evidenced by the overlap between I 5p and N 2p orbitals in the I_2_/ImPh system (Figure 2b). This hybridization indicates direct involvement of the imine sites in iodine binding. Consistently, the total density of states (TDOS) reveals a clear modification of the electronic structure after iodine adsorption, accompanied by a reduction in the band gap (Figure S4a), suggesting enhanced electronic delocalization within the framework. The PDOS analysis further shows a shift of the nitrogen p orbital to lower energy upon interaction with iodine (Figure S4b), indicating an electron density redistribution at the imine sites. These results reveal that iodine adsorption perturbs the electronic structure of imine sites, enabling tunable iodine–framework interactions.

The charge‐density distribution of HImPh reveals localized electron density around the imine sites, indicating that they are proton‐switchable centers (Figure 2c). In contrast, the ImPh framework exhibits a more delocalized electronic distribution along the conjugated backbone (Figure S5), indicating that protonation redistributes electron density and alters the local electronic environment at the adsorption sites. The charge density difference further reveals the distinct interaction modes of iodine species on ImPh and HImPh. For I_3_^−^ adsorption on ImPh, pronounced electron accumulation and depletion regions are distributed along the conjugated backbone and the adsorbed polyiodide chain, indicating strong delocalized electronic coupling between the framework and extended iodine species (Figure 2d). This delocalization facilitates stabilization of polyiodide species along the π‐conjugated structure. In contrast, I^−^ adsorption on HImPh is characterized by highly localized charge redistribution around the protonated imine site, with electron density concentrated near the adsorption center (Figure 2e). This localized interaction reflects strong electrostatic stabilization of I^−^ at the protonated site, rather than extended coupling with the framework, arising from protonation‐induced charge localization that converts the neutral imine into a positively polarized adsorption center. Similar trends are observed for other iodine adsorption configurations (Figure S6), further supporting the protonation‐dependent modulation of iodine–framework interaction. Although adsorption of neutral I_2_ remains favorable in both states due to polarization interactions with the conjugated framework, protonation markedly redistributes adsorption preferences among charged iodine species. Neutral ImPh preferentially stabilizes extended polyiodides, whereas protonated HImPh favors localized iodide adsorption through enhanced electrostatic interaction.

Such proton‐switching behavior predicted by theory is directly supported by spectroscopic evidence. The ex situ ATR‐FTIR spectra show the emergence of a characteristic C═NH^+^ vibration in the imine region during discharge, while its intensity decreases upon charging, indicating reversible protonation and deprotonation of the imine sites (Figure 2f) [21, 22]. Ex situ N 1s x‐ray photoelectron spectroscopy (XPS) further reveals a corresponding evolution of nitrogen chemical states during electrochemical cycling (Figure 2g). The discharged electrode exhibits a pronounced C═NH^+^ component [23, 24], consistent with protonation of imine sites accompanying polyiodide reduction during discharge (I_3_^−^ + 2e^−^ → 3I^−^). Upon charging, oxidation of iodide to polyiodides (3I^−^ → I_3_^−^ + 2e^−^) is accompanied by deprotonation of the imine sites, as reflected by the diminished C═NH^+^ feature, confirming the reversibility of the proton‐switching process. The corresponding quantitative fitting of N 1s spectra (Figure S7a) shows a significant increase in the fraction of protonated imine species during discharge, which recovers upon charging. Notably, the overall N signal remains preserved throughout cycling, indicating that the framework undergoes reversible proton switching without structural degradation. Together, these results confirm reversible protonation of imine sites as the origin of the proton‐switching mechanism (Figure 2h). This process should be understood as a local interfacial proton‐coupled electronic modulation under electrochemical polarization, rather than a bulk pH‐driven transformation. Consistently, pH‐dependent zeta‐potential measurements (Figure S7b) show systematic variations in surface potential, reflecting the intrinsic proton responsiveness of the framework. This behavior supports the feasibility of proton switching, while the electrochemical process is governed by dynamic interfacial reactions rather than bulk pH variation. Thus, these results provide direct experimental evidence for a dynamic proton‐switching mechanism governing iodine–framework interactions.

To directly probe the evolution of iodine species during electrochemical cycling, in situ UV–vis and Raman spectroscopy were performed on ImPh and Ketjen Black (KB) electrodes. During charging, the electrolyte in contact with ImPh exhibits only weak absorption features associated with polyiodide species, suggesting that polyiodide intermediates are mainly confined within the ImPh framework rather than released into the bulk electrolyte (Figure 3a). In contrast, the KB‐based system shows pronounced, continuously increasing absorption bands corresponding to I_3_^−^, accompanied by a visible darkening of the electrolyte (Figure 3b), suggesting substantial polyiodide dissolution. The divergence between the two systems becomes more evident during discharge. The electrolyte from the KB cell retains strong polyiodide‐related absorption, whereas the ImPh‐based electrolyte shows only negligible signals throughout the process (Figure 3c), indicating rapid consumption and suppressed persistence of polyiodide species on ImPh.

**FIGURE 3:**
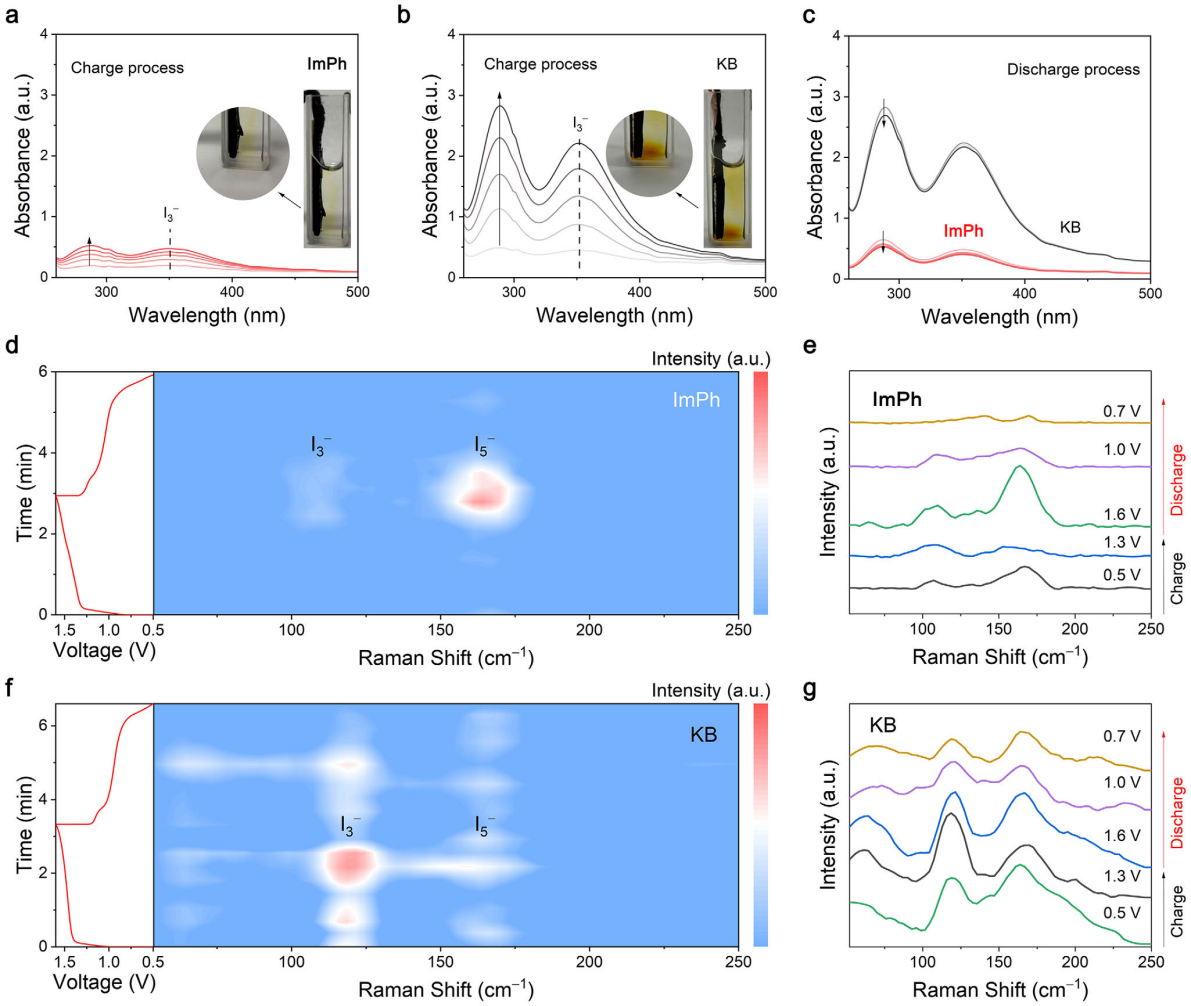
Proton‐switch‐regulated polyiodide conversion in Zn–I_2_ batteries. In situ UV–vis absorption spectra of the electrolytes during the charging process for (a) ImPh‐based and (b) KB‐based cathodes, with corresponding photographs of the electrolyte. (c) Evolution of polyiodide species in the electrolyte during the discharge process for ImPh‐based and KB‐based cathodes. In situ Raman contour map and representative Raman spectra of (d, e) ImPh‐based and (f, g) KB‐based full batteries during charge and discharge.

In situ Raman measurements further reveal the dynamic evolution of iodine species during cycling. For the ImPh electrode, polyiodide signals emerge only transiently during the charging process, with weak I_3_^−^ (∼110 cm^−1^) and short‐lived I_5_^−^ (∼150–170 cm^−1^) features appearing within a limited potential window, and rapidly disappearing upon discharge, indicating fast conversion without accumulation (Figures 3d,e). In contrast, the KB electrode exhibits strong and persistent Raman signals from both I_3_^−^ and I_5_^−^ that develop during charging and persist throughout discharge (Figures 3f,g), indicating continuous formation and retention of polyiodides. This contrast demonstrates fundamentally different iodine reaction pathways: ImPh confines polyiodides as short‐lived intermediates, whereas KB enables their sustained accumulation, indicating a reduced residence time of polyiodide intermediates at the interface.

To clarify whether proton‐switching regulation alters iodine redox kinetics, a comprehensive kinetic analysis was performed on ImPh and KB electrodes. In situ electrochemical impedance spectra (EIS) reveal that ImPh exhibits consistently lower charge‐transfer resistance throughout the charge–discharge process than KB, indicating faster interfacial kinetics under dynamic operation (Figure S8). Cyclic voltammetry (CV) curves further reveal higher current responses with reduced polarization for ImPh (Figures 4a and S9a). The derived b‐values approach unity for both anodic and cathodic processes (Figure 4b), indicating a dominant pseudocapacitive‐controlled mechanism compared with KB (Figure S9b). Quantitative analysis further shows a higher capacitive contribution over the entire scan‐rate range (Figures 4c and S9c), suggesting that iodine conversion is governed by fast interfacial processes rather than diffusion‐limited behavior. Consistently, galvanostatic intermittent titration technique (GITT) analysis reveals comparable diffusion behavior for ImPh and KB (Figures 4d and S9d), with similar apparent diffusion coefficients of iodine species on the order of 10^−10^ cm^2^ s^−1^. This result indicates that the introduction of ImPh imposes no additional diffusion limitation, suggesting that iodine regulation is dominated by interfacial conversion rather than bulk diffusion processes.

**FIGURE 4:**
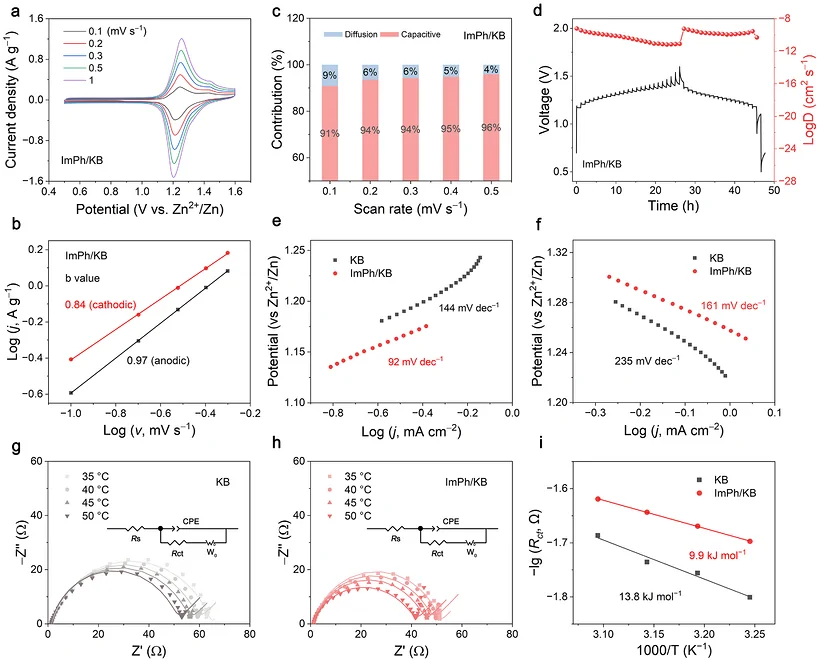
Kinetic analysis of iodine redox on ImPh/KB electrodes. (a) CV curves of the ImPh/KB electrode at different scan rates. (b) Determination of *b* values derived from the relationship between peak current and scan rate for anodic and cathodic processes. (c) Quantitative separation of diffusion‐controlled and capacitive contributions at different scan rates. (d) GITT profile of the ImPh/KB electrode with the calculated apparent diffusion coefficients. (e, f) Tafel plots of KB and ImPh/KB electrodes derived from polarization curves. (g, h) Nyquist plots of KB and ImPh/KB electrodes measured at different temperatures. (i) Arrhenius plots for KB and ImPh/KB‐based Zn‐I_2_ batteries.

Furthermore, Tafel analysis reveals a substantially reduced slope for the ImPh electrode (92 mV dec^−1^) compared with KB (144 mV dec^−1^), indicating accelerated iodine‐redox kinetics enabled by proton‐switch‐regulated interfacial conversion (Figure 4e). Similar behavior is observed for the anodic process, where ImPh also exhibits a smaller oxidation Tafel slope than KB (Figure 4f), suggesting facilitated polyiodide oxidation kinetics. Temperature‐dependent EIS measurements further reveal consistently lower charge‐transfer resistance for ImPh over the entire temperature range from 35°C to 50°C (Figure 4g,h; Tables S1 and S2). Arrhenius analysis yields a lower apparent activation energy for the ImPh electrode (9.9 kJ mol^−1^) than for KB (13.8 kJ mol^−1^) (Figure 4i), indicating reduced kinetic barriers for interfacial iodine conversion. These results collectively demonstrate that proton‐switching regulation not only suppresses polyiodide accumulation but also preserves rapid iodine‐redox kinetics through dynamically regulated interfacial interactions.

The impact of the proton‐switching mechanism is ultimately manifested in the electrochemical performance of the Zn–I_2_ battery system. The ImPh‐based full cell maintains well‐defined discharge plateaus with minimal polarization over a wide current density range from 0.5 to 20 A g^−1^ (Figure 5a), indicating that rapid iodine conversion is preserved despite the suppression of polyiodide accumulation. Moreover, the ImPh‐based cell consistently delivers higher reversible capacities than the KB counterpart across all current densities, and the capacity is largely recovered upon returning to low rates, indicating stable reaction reversibility (Figure 5b). At a low current density of 0.2 A g^−1^, the ImPh‐based cell exhibits stable cycling with a high average CE of 96.9%, whereas the KB electrode shows continuous capacity decay with a lower average CE of 90.6% (Figures 5c and S10). More notably, under high‐rate operation at 20 A g^−1^, ImPh sustains stable cycling over 51 000 cycles with negligible capacity loss and a CE close to 100% (Figures 5d and S11). In contrast, the KB control cell exhibits a slightly lower average CE of approximately 99.7% together with more pronounced capacity decay, while the CyVi‐based Zn–I_2_ cell without proton‐switching sites also undergoes rapid capacity fading under the same condition (Figure S12). These comparisons confirm that the superior long‐term stability of ImPh cannot be attributed simply to carbon conductivity, pore confinement, or the conjugated framework, but arises from proton‐switching‐regulated iodine chemistry. In addition, the iodine‐free ImPh/KB cathode delivers only a low capacity of approximately 24 mAh g^−1^ (Figure S13), confirming that the long‐term capacity mainly originates from reversible iodine redox rather than the intrinsic capacity contribution of the ImPh framework.

**FIGURE 5:**
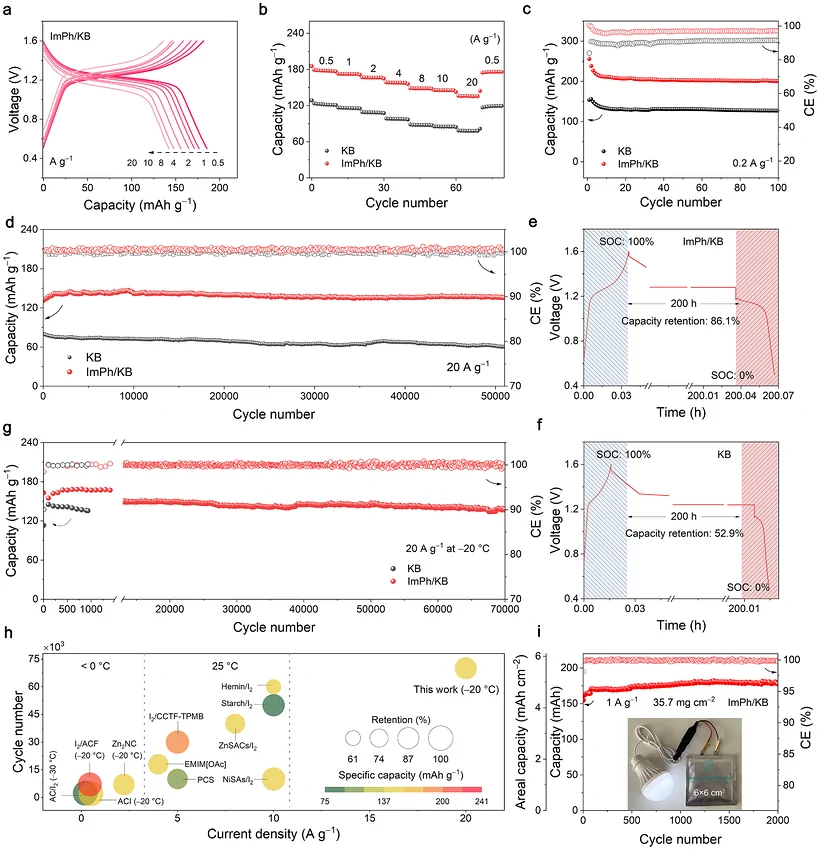
Electrochemical performance of ImPh‐based Zn–I_2_ batteries. (a) Galvanostatic charge–discharge profiles of the ImPh/KB cell at different current densities. (b) Rate capability comparison between KB and ImPh/KB electrodes. (c) Cycling stability at 0.2 A g^−1^ with corresponding CE. (d) Long‐term cycling performance at 20 A g^−1^ at room temperature. Self‐discharge performance of Zn–I_2_ batteries with (e) ImPh/KB and (f) KB cathodes. (g) Ultralong cycling stability at 20 A g^−1^ under −20°C conditions. (h) Comparison of electrochemical performance with representative Zn–I_2_ cathodes reported in the literature. (i) Cycling performance of a high‐loading ImPh‐based pouch cell at 1 A g^−1^.

After prolonged cycling, both FTIR and XRD characterizations show that the characteristic framework vibrations and diffraction feature of ImPh are retained, confirming the chemical and structural durability of the framework during long‐term electrochemical operation (Figure S14). Meanwhile, XRD patterns of cycled Zn anodes reveal suppressed by‐product formation in the ImPh‐based system compared with KB (Figure S15), suggesting mitigated parasitic reactions. Consistently, SEM images show a smooth and uniform Zn surface after cycling in the ImPh cell, whereas severe corrosion and dendritic features are observed for the KB counterpart (Figure S16), indicating that regulated iodine chemistry effectively suppresses shuttle‐induced anode degradation. In addition, self‐discharge measurements reveal markedly improved capacity retention for the ImPh‐based cell, delivering 94% retention after 48 h and 86.1% after 200 h of prolonged resting, substantially outperforming the KB counterpart (52.9%) (Figures 5e,f, and S17). This sustained retention over an extended time evidences effective suppression of polyiodide shuttling and enhanced interfacial stability. Benchmarking against representative Zn–I_2_ systems further highlights the superior self‐discharge resistance achieved in this work (Table S3).

To evaluate performance under harsh conditions, the proton‐switching mechanism was further examined in low‐temperature electrolytes, where it remained operative (Figure S18). At −20°C, the ImPh‐based cell maintains stable cycling up to 70 000 cycles, while the KB‐based battery rapidly fails after 930 cycles (Figure 5g). Benchmark comparison shows that the ImPh system delivers one of the longest cycle lives among reported Zn–I_2_ batteries under subzero conditions and at high current densities (Figure 5h and Table S4) [13, 17, 25, 26, 27, 28, 29, 30, 31, 32, 33, 34]. Furthermore, a high‐loading cathode (35.7 mg cm^−2^) achieves stable cycling at 1 A g^−1^ with sustained areal capacity and an active‐iodine‐based gravimetric energy density of approximately 120 Wh kg^−1^ (Figure 5i), demonstrating the scalability of the proton‐switching strategy beyond thin‐electrode configurations. A comparison with representative Zn–I_2_ pouch cell systems reported in the literature highlights the superior combination of high iodine loading, areal capacity, and cycling durability achieved in this work (Table S5) [9, 11, 17, 35, 36, 37, 38, 39, 40, 41]. Collectively, these results show that proton‐switching regulation effectively suppresses shuttle‐induced parasitic reactions while maintaining rapid iodine conversion kinetics across practical operating conditions, including high rates, low temperatures, and high iodine loadings.

To further examine whether proton‐switching behavior is generally applicable to other imine‐linked frameworks, two reported imine‐linked 2D polymer frameworks with distinct backbone structures, denoted as ImPh‐2 and ImPh‐3, were selected as model systems (Figure S19) [42, 43]. These two frameworks contain analogous imine linkages but differ in their molecular backbones, allowing us to evaluate the generality of the proton‐switching mechanism beyond the ImPh structure. Both ImPh‐2 and ImPh‐3 exhibit characteristic reversible changes in imine‐related FTIR vibrations during charge and discharge, indicating that protonation–deprotonation behavior is not unique to ImPh but can occur more broadly in imine‐linked 2D frameworks. In addition, both frameworks exhibit reduced charge‐transfer resistance and improved iodine‐redox kinetics compared with KB electrodes in Zn–I_2_ batteries. These observations further support that the proton‐switching mechanism arises from reversible electronic modulation of imine sites within structurally defined frameworks.

## 3 Conclusion

In summary, we establish a proton‐switch‐regulated mechanism that dynamically modulates iodine speciation through reversible electronic control of imine sites within a 2D polymer framework. This state‐dependent interfacial regulation switches iodine–framework interactions between I^−^ stabilization during discharge and polyiodide stabilization during charge, enabling adaptive control over soluble iodine intermediates without sacrificing reaction kinetics. As a result, Zn–I_2_ batteries deliver ultralong cycling stability of 51 000 cycles at 20 A g^−1^ at room temperature and over 70 000 cycles at −20°C. Notably, this performance is retained under practical conditions, where high iodine loadings (35.7 mg cm^−2^) achieve areal capacities of 5 mAh cm^−2^ over 2000 cycles with negligible decay. This work establishes a state‐dependent interfacial regulation paradigm that overcomes the intrinsic limitations of static confinement and provides a general framework for controlling soluble redox intermediates in aqueous electrochemical systems.

## Author Contributions

**Feifei Wang**: conceptualization, methodology, formal analysis, validation, investigation, writing – review and editing, writing – original draft, data curation. **Guoqin Liu**: investigation, data curation, formal analysis, validation. **Yuhang Zhuang**: investigation, resources, formal analysis. **Peng Zhang**: investigation, formal analysis. **Zhengwei Li**: formal analysis, investigation. **Songshan Bi**: data curation, formal analysis. **Ahmad Bagheri**: data curation, validation. **Angelika Wrzesińska‐lashkova**: validation. **Neda Kargarfard**: investigation. **Peyman Rostami**: investigation. **Yana Vaynzof**: resources, writing – review and editing. **Bernd Plietker**: writing – review and editing. **Mingchao Wang**: writing – review and editing. **Ali Shaygan Nia**: writing – review and editing, supervision, funding acquisition. **Xinliang Feng**: writing – review and editing, supervision, project administration, funding acquisition.

## Conflicts of Interest

The authors declare no conflicts of interest.

## Supporting information




**Supporting File**: anie74010‐sup‐0001‐SuppMat.docx.

## Data Availability

The data that support the findings of this study are available from the corresponding author upon reasonable request.
