# Supplementary material for: Proton‐Switching Regulates Interfacial Iodine Chemistry for Long‐Life Zinc–Iodine Batteries
Source: Angew Chem Int Ed Engl. 2026 Aug 5;65(40):e4465475. doi: 10.1002/anie.4465475 (PMC13618271; doi:10.1002/anie.4465475)
Supplement: Supplementary file 1 [file ANIE-65-e4465475-s001.docx]

**Supporting Information:**

**Proton‐Switching Regulates Interfacial Iodine Chemistry for Long-Life Zinc–Iodine Batteries**

*Feifei Wang**^1,2^, Guoqin Liu^1,2^, Yuhang Zhuang^2^, Peng Zhang^1^,* *Zhengwei Li^3^, Songshan Bi^2^, Ahmad Bagheri^1^,* *Angelika Wrzesińska-Lashkova^4,5^, Neda Kargarfard^1^, Peyman Rostami^6^, Yana Vaynzof^4,5^, Bernd Plietker^3^, Mingchao Wang^1,7^, Ali Shaygan Nia^1,2^** *& Xinliang Feng^1,2^**

^1^ Max Planck Institute for Microstructure Physics, 06120 Halle (Saale), Germany

^2^ Center for Advancing Electronics Dresden (cfaed) and Faculty of Chemistry and Food Chemistry, Technische Universität Dresden, 01062 Dresden, Germany

^3^ Chair of Organic Chemistry I, Faculty of Chemistry and Food Chemistry, Technische Universität Dresden, 01069 Dresden, Germany

^4^ Chair for Emerging Electronic Technologies, Technische Universität Dresden, 01062 Dresden, Germany

^5^ Leibniz Institute for Solid State and Materials Research Dresden, 01069 Dresden, Germany

^6^ Leibniz Institute of Polymer Research Dresden (IPF), Institute of Physical Chemistry and Polymer Physics, 01069 Dresden, Germany

^7^ State Key Laboratory of Advanced Waterproof Materials & Guangdong Provincial Key Laboratory of Nano-Micro Materials Research, School of Advanced Materials, Peking University, Shenzhen Graduate School, Shenzhen, China

*E-mail: ali.shaygan_nia@mpi-halle.mpg.de, xinliang.feng@mpi-halle.mpg.de

*^#^*Feifei Wang, Guoqin Liu, and Yuhang Zhuang contribute equally to this work.

**Experimental sections**

**Chemicals and Materials**

Iodine (I₂, >99.99%), potassium iodide (KI, A.R.), zinc sulfate heptahydrate (ZnSO₄·7H₂O, A.R.), ethanol, and sulfuric acid were purchased from commercial suppliers and used as received. Zinc foil (100 μm thickness), polytetrafluoroethylene (PTFE) binder, and Ketjen black (KB) were obtained from Canrd Technology Co., Ltd.

**ImPh synthesis**

A glass ampule was charged with [1,1′:3′,1″-terphenyl]-4,4″,5′-tricarbaldehyde (15 mg, 47.8 μmol, 1.0 equiv), benzene-1,4-diamine (7.7 mg, 71.6 μmol, 1.5 equiv), and 6 M acetic acid (0.1 mL) in a mixed solvent of mesitylene and 1,4-dioxane (0.2/0.8 mL). The mixture was sonicated at room temperature for 10 min, degassed by three freeze–pump–thaw cycles, flame-sealed under vacuum, and heated at 120 °C for 3 days. After cooling to room temperature, the resulting precipitate was collected by filtration and washed successively with DMF, THF, H_2_O, ethanol, and acetone. The product was dried under vacuum at 100 °C overnight to get the ImPh powder.

**Electrode and Electrolyte Preparation**

The ImPh-based cathode was prepared by mixing KB and ImPh with PTFE binder in a mass ratio of 7:2:1 using ethanol as the dispersion solvent. The resulting slurry was uniformly pressed onto carbon cloth current collectors and dried under vacuum at 60 °C overnight. A control cathode was fabricated using KB and PTFE in a mass ratio of 9:1 following the same procedure. For iodine adsorption and spectroscopic measurements, iodine-containing solutions were prepared by dissolving I_2_ (or including LiI) in deionized water at designated concentrations.

**Electrochemical Measurements**

Zn–I_2_ batteries were assembled in CR2032 coin cells using Zn foil as the anode, ImPh-based or KB-based electrodes as the cathode, and cellulose membrane as the separator. Typically, 10 μL of cathodic electrolyte was added to each cell. Unless otherwise specified, the electrolyte consisted of a 2 M ZnSO_4_ aqueous solution. For low-temperature electrochemical measurements, a 3 M Zn(ClO_4_)_2_ aqueous electrolyte was used to improve electrolyte stability under subzero conditions. Galvanostatic charge–discharge cycling was conducted using LAND (CT3002A) testing systems. Specific capacities were calculated based on the mass of iodine. Cyclic voltammetry and EIS measurements were performed on a CHI 660E electrochemical workstation. EIS spectra were collected in the frequency range of 0.1–10^5^ Hz with an AC amplitude of 5 mV. All electrochemical measurements were carried out at 25 °C, unless otherwise noted. For high-loading pouch cells, ImPh-containing KB was ground and placed in a glass vial, which was then sealed in a stainless-steel reactor (Anhui Kemi Instrument Co., Ltd.) with solid iodine placed at the bottom. Iodine was incorporated into the ImPh/KB composite by room-temperature vapor diffusion. The resulting iodine-loaded composite was mixed with PTFE to fabricate cathode and assembled into rectangular pouch cells (6 × 6 cm^2^) with iodine loadings up to 35.7 mg cm^−2^ on both sides.

**Structural characterization**

XRD patterns were recorded on a Bruker D8 using Cu Kα radiation. SEM and EDS were performed to examine morphology and elemental distribution. XPS measurements were carried out on a Thermo Fisher Scientific spectrometer. All binding energies were calibrated using the C–C peak at 284.8 eV. UV–visible absorption spectra were collected using a UV–vis spectrophotometer equipped with quartz cuvettes (optical path length: 1 cm). The concentration of iodine species was adjusted to ensure measurements within the linear absorbance range. Raman spectroscopy was conducted on a confocal Raman microscope using a 532 nm laser. *In situ* Raman measurements were performed during galvanostatic charge–discharge cycling to monitor the evolution of iodine species in real time. Zeta potential measurements were conducted using a dynamic light scattering instrument. ImPh powders were dispersed in aqueous solutions with controlled pH values adjusted by dilute sulfuric acid.

**DFT calculations**

Adsorption energy calculations were carried out using the Vienna Ab initio Simulation Package (VASP) within the framework of density functional theory. The electron–ion interaction was described by the projector augmented wave (PAW) method, and the exchange–correlation effects were treated using the Perdew–Burke–Ernzerhof (PBE) functional within the generalized gradient approximation (GGA) [1–3]. The cutoff energy for the plane-wave basis set was set to 500 eV. A Γ-centered 3 × 3 × 1 k-point mesh was used for Brillouin zone sampling. The electronic self-consistent convergence criterion was set to 1 × 10^−5^ eV. Structural relaxations were performed using the conjugate gradient algorithm until the residual force on each atom was less than 0.02 eV Å^−1^. van der Waals interactions were accounted for using the DFT-D3 method with Becke–Johnson damping. Model construction, input generation, and post-processing of the VASP results were performed using the VASPKIT package [4]. The adsorption energy (E_ads) was defined as:

$E_{ads}=E_{total}-E_{substrate}-E_{adsorbate}$

**Figure S1**. **Structural and morphological characterization of ImPh.** (a) Powder XRD pattern of ImPh and the simulated structure. (b) N_2_ adsorption–desorption isotherms of ImPh. (c) SEM image of ImPh particles; (d) SEM image and elemental mapping of N and C.

**Figure S2.** UV–vis analysis of I_3_^−^ adsorption by ImPh. (a) UV–vis absorption spectra of I_3_^−^ aqueous solutions at different concentrations. (b) Linear relationship between absorbance and I_3_^−^ concentration. (c) UV–vis spectra of I_3_^−^ solution before and after contact with ImPh.

**Figure S3**. Powder XRD pattern of the I_2_/ImPh composite.

**Figure S4. DOS analysis of ImPh before and after iodine adsorption.** (a) TDOS of ImPh and I_2_/ImPh. (b) PDOS of N p orbitals before and after I_2_ adsorption.

**Figure S5**. Charge density distribution of the deprotonated ImPh framework.

**Figure S6. Charge density difference for iodine adsorption on ImPh and HImPh.** (a) ImPh···I^−^ configuration. (b) ImPh···I_2_ configuration. (c) HImPh···I_2_ configuration. (d) HImPh···I_3_^−^ configuration (Yellow and cyan regions represent electron accumulation and depletion, respectively).

In Figure S6, charge density difference analyses for additional adsorption configurations, including I⁻ and I₂ on ImPh and HImPh, as well as I_3_^−^ on HImPh, are presented. For deprotonated ImPh, electron redistribution is generally delocalized along the conjugated framework, consistent with extended interaction with iodine species. In contrast, protonated HImPh exhibits more localized charge redistribution centered around the imine sites, regardless of the iodine species. These observations are consistent with the main-text analysis and further confirm that protonation alters the interaction mode from delocalized coupling to localized electrostatic stabilization.

**Figure S7.** (a) Atomic ratio of imine (C=N) and protonated imine (C=NH^+^) species derived from N 1s XPS spectra at different electrochemical states (discharge–charge–discharge). (b) pH-dependent zeta potential of ImPh.

**Figure S8. *In situ* electrochemical impedance spectroscopy during charge–discharge process.** (a) ImPh/KB-based Zn–I_2_ cell. (b) KB-based Zn–I_2_ cell.

**Figure S9. Electrochemical kinetic analysis of KB-based Zn–I_2_ cells.** (a) Cyclic voltammetry curves at different scan rates. (b) Relationship between peak current and scan rate with derived b values. (c) capacitive and diffusion-controlled contributions at different scan rates. (d) GITT curves measured at 0.1 A g^−1^.

**Figure S10.** (a) Galvanostatic charge–discharge profiles of the KB-based cell at different current densities. Representative voltage profiles at the 1st and 100^th^ cycles based on different cathodes. (b) ImPh/KB and (c) KB.

**Figure S11.** Long-term cycling stability evaluation of the ImPh/KB-based Zn–I_2_ cell. (a) Charge–discharge profiles at the 1,000^th^ and 51,000^th^ cycles. (b) EIS spectra before and after long-term cycling.

**Figure S12.** Electrochemical evaluation of the CyVi control without proton-switching sites. (a) Chemical structure of the CyVi framework. (b) Cycling performance and CE of the CyVi/KB-based Zn–I_2_ cell without proton-switching sites at 20 A g^−1^.

**Figure S13.** Cycling performance and Coulombic efficiency of the ImPh/KB-based cathode without iodine active material at 20 A g^−1^.

**Figure S14.** Post-cycling structural characterization of ImPh. (a) Powder XRD pattern and (b) FTIR spectrum of ImPh after 1000 electrochemical cycles.

**Figure S15.** XRD patterns of Zn anodes after 1000 electrochemical cycles.

**Figure S16. SEM images of Zn anodes after cycling in Zn–I_2_ cells.** (a,b) Zn anode from the ImPh-based cell at different magnifications. (c,d) Zn anode from the KB-based cell at different magnifications.

**Figure S17.** Self-discharge performance of Zn–I_2_ batteries with ImPh/KB cathodes (48 h).

**Figure S18.** Spectroscopic characterization of ImPh under different electrochemical states at −20 °C. (a) ATR-FTIR spectra; (b) N 1s XPS spectra; (c) atomic ratio of C=N and C=NH^+^ species.

**Figure S19.** **Generality of the proton-switch mechanism in additional imine-linked 2D polymer frameworks.** Structural models of (a) ImPh-2 and (b) ImPh-3. *Ex-situ* FTIR spectra of (c) ImPh-2 and (d) ImPh-3 collected at pristine, charged, and discharged states. (e) Nyquist plots of KB, ImPh-2, and ImPh-3 based Zn-I_2_ batteries before cycling. (f) CV curves of KB, ImPh-2, and ImPh-3 based Zn-I_2_ batteries.

These results indicate that proton-switch-regulated iodine chemistry represents a transferable interfacial regulation principle enabled by reversible electronic modulation of imine-linked frameworks.

**Table S1**. Fitting results of *R*_ct_ at different temperatures for the ImPh-based Zn–I_2_ batteries.

| Temperature | 35 ℃ | 40 ℃ | 45 ℃ | 50 ℃ |
| --- | --- | --- | --- | --- |
| *R*_ct_ (Ω) | 49.8 | 46.7 | 44 | 41.6 |
| Error (%) | 3.8 | 2.9 | 3.8 | 1.6 |

**Table S2**. Fitting results of *R*_ct_ at different temperatures for the KB-based Zn–I_2_ batteries.

| Temperature | 35 ℃ | 40 ℃ | 45 ℃ | 50 ℃ |
| --- | --- | --- | --- | --- |
| *R*_ct_ (Ω) | 63.2 | 57 | 54.4 | 48.6 |
| Error (%) | 2.9 | 2.6 | 2.6 | 4.2 |

**Table S3**. Comparison of self-discharge performance of representative Zn–I_2_ batteries reported in the literature and this work.

| Cathode | Testing time (h) | Capacity retention (%) | References |
| --- | --- | --- | --- |
| **ImPh** | **48** | **94** | **This work** |
| **ImPh** | **200** | **86.1** | **This work** |
| AC@I_2_ | 24 | 75 | [5] |
| I_2_/PPCMK | 168 | 81.72 | [6] |
| AC/ZnI2 | 168 | 61.73 | [7] |
| AC/ZnI2 | 48 | 93.53 | [8] |
| ZnI2/CP | 48 | 90.2 | [9] |
| HC@FeNC | 24 | 95.52 | [10] |
| AC@I_2_ | 48 | 90.89 | [11] |
| AC@I_2_ | 48 | 85 | [12] |

**Table S4.** Comparison of electrochemical performance of representative Zn–I_2_ batteries reported in the literature and in this work, including current density, specific capacity, capacity retention, cycle life, and operating temperature.

| Cathode | j (A g^‒1^) | Capacity (mAh g^‒1^) | Capacity retention (%) | Cycle number | T (℃) | References |
| --- | --- | --- | --- | --- | --- | --- |
| **ImPh** | **20** | **143.4** | **95** | **70000** | **−20** | **This work** |
| **ImPh** | **20** | **139.4** | **~100** | **51000** | **25** | **This work** |
| ZnSACs | 8 | 150 | 80.5 | 40000 | 25 | [13] |
| EMIM[OAc] | 4 | 149.23 | 82 | 18000 | 25 | [11] |
| PCS hydrogel | 5 | 102 | 82.95 | 10000 | 25 | [14] |
| Pyridine additive | 10 | 105.5 | 84 | 27500 | 25 | [15] |
| I_2_/NiSAs | 10 | 151 | 94 | 10000 | 25 | [16] |
| Starch | 10 | 75 | 90 | 50000 | 25 | [17] |
| I_2_/CCTF-TPMB | 5 | 180 | 93.9 | 30000 | 25 | [18] |
| Hemin | 10 | 148 | 61 | 60000 | 25 | [19] |
| AC/I_2_ | 0.2 | 89 | 100 | 2300 | **−**30 | [20] |
| Zn_2_NC | 2.21 | 140 | 87 | 7000 | **−**20 | [21] |
| ACI | 0.5 | 140 | 100 | 2000 | **−**20 | [22] |
| I_2_/ACF | 0.44 | 200 | 97 | 7000 | **−**20 | [23] |

**Table S5.** Comparison of pouch-cell performance of aqueous Zn–I_2_ batteries reported in recent literature and this work, including iodine loading, areal capacity, cycle life, and capacity retention at room temperature.

| Cathode | Mass loading (mg cm^‒2^) | Areal capacity (mAh cm^‒2^) | Capacity retention (%) | Cycle number | References |
| --- | --- | --- | --- | --- | --- |
| **ImPh** | **35.7** | **5** | **~100** | **2000** | **This work** |
| Hemin | 3.4 | 0.38 | 93 | 500 | [19] |
| Zn_1_Co/I_2_ | 30 | 6.6 | 50 | 240 | [24] |
| S-3@I_3_^−^ | 2.8 | 0.35 | 82 | 120 | [25] |
| AC/I_2_ | 31.3 | 5 | 90.6 | 50 | [26] |
| AC/I_2_ | 10 | 4.6 | 71.4 | 400 | [27] |
| SAZn@CFS | 15.5 | 1.6 | 100 | 1100 | [28] |
| KB-PAC/I_2_ | 15 | - | 91.38 | 1000 | [29] |
| BMT | 15.5 | 1.8 | 76.2 | 800 | [30] |
| Super P/I_2_ | 12.8 | 2.2 | 80 | 500 | [31] |
| Co@CNF layer | 16 | 2.7 | 90 | 550 | [32] |

**References**

1.	G. Kresse, and J. Furthmüller, “Efficient iterative schemes for *ab initio* total-energy calculations using a plane-wave basis set,” *Physical Review B*, 54 (1996): 11169–11186, https://doi.org/10.1103/PhysRevB.54.11169.

2.	P. Hohenberg, and W. Kohn, “Inhomogeneous electron gas,” *Physical Review*, 136 (1964): B864–B871, https://doi.org/10.1103/PhysRev.136.B864.

3.	J. P. Perdew, K. Burke, and M. Ernzerhof, “Generalized gradient approximation made simple,” *Phys. Rev. Lett.*, 77 (1996): 3865–3868, https://doi.org/10.1103/PhysRevLett.77.3865.

4.	V. Wang, N. Xu, J.-C. Liu, G. Tang, and W.-T. Geng, “VASPKIT: a user-friendly interface facilitating high-throughput computing and analysis using VASP code,” *Computer Physics Communications*, 267 (2021): 108033, https://doi.org/10.1016/j.cpc.2021.108033.

5.	B. Niu, X. Jian, Z. Hu, P. Wang, and X. Wang, “Thermal phase transition electrolyte for high‐temperature aqueous zinc‐metal batteries,” *Advanced Materials*, 38 (2025): e14164, https://doi.org/10.1002/adma.202514164.

6.	S. Zeng, S. Chen, Z. Ao, X. Lin, L. Yan, C. Liu, and Z. Lin, “Dual‐mechanism anchoring of iodine species by pitch‐derived porous carbon for enhanced zinc–iodine battery performance,” *Small*, 21 (2025): 2501695, https://doi.org/10.1002/smll.202501695.

7.	H. Wu, S.-J. Zhang, J. Vongsvivut, M. Jaroniec, J. Hao, and S.-Z. Qiao, “Aqueous zinc-iodine batteries with ultra-high loading and advanced performance,” *Joule*, 9 (2025): 102000, https://doi.org/10.1016/j.joule.2025.102000.

8.	X. Huang, T. Pan, B. Zhang, J. Wang, T. Hu, A. Duan, S. Luo, B. Zhao, M. Li, Y. Lin, and W. Sun, “Functionally segregated ion regulation enables dual confinement effect for highly stable zinc‐iodine batteries,” *Advanced Materials* (2025): 2500500, https://doi.org/10.1002/adma.202500500.

9.	J.-L. Yang, T. Xiao, T. Xiao, J. Li, Z. Yu, K. Liu, P. Yang, and H. J. Fan, “Cation‐Conduction Dominated Hydrogels for Durable Zinc–Iodine Batteries,” *Advanced Materials* (2024): 2313610, https://doi.org/10.1002/adma.202313610.

10.	Y. Wang, X. Jin, J. Xiong, Q. Zhu, Q. Li, R. Wang, J. Li, Y. Fan, Y. Zhao, and X. Sun, “Ultra‐stable Electrolytic Zn‐I_2_ Batteries Based on Nanocarbon Wrapped by Highly Efficient Single‐Atom Fe‐NC Iodine Catalysts,” *Advanced Materials* (2024): 2404093, https://doi.org/10.1002/adma.202404093.

11.	T. Xiao, J. Yang, B. Zhang, J. Wu, J. Li, W. Mai, and H. J. Fan, “All‐Round Ionic Liquids for Shuttle‐Free Zinc‐Iodine Battery,” *Angewandte Chemie International Edition*, 63 (2024): e202318470, https://doi.org/10.1002/anie.202318470.

12.	S. Cao, A. Zhang, H. Fang, B. Feng, Y. Liu, P. Yi, S. He, Z. Ren, L. Ma, W. Lu, M. Ye, and J. Shen, “Skin-like quasi-solid-state electrolytes for spontaneous zinc-ion dehydration toward ultra-stable zinc–iodine batteries,” *Energy & Environmental Science*, 18 (2025): 3395–3406, https://doi.org/10.1039/D4EE05527F.

13.	Y. Zhao, Y. Wang, W. Xue, H. Huang, J. Li, C. Hu, and D. Liu, “Elucidating synergistic mechanism of zinc single‐atom sites and lewis acid–base pairs to boost zinc–iodine batteries performance,” *Advanced Functional Materials*, 35 (2025): 2422677, https://doi.org/10.1002/adfm.202422677.

14.	J. Li, C. Li, J. Ren, P. Li, K. Zhang, T. Wu, and L. Wang, “Synergistic effect of organic-inorganic hybrid electrolyte for ultra-long Zn–I_2_ batteries,” *International Journal of Hydrogen Energy*, 48 (2023): 21985–21995, https://doi.org/10.1016/j.ijhydene.2023.03.041.

15.	Y. Lyu, J. Yuwono, P. Wang, Y. Wang, F. Yang, S. Liu, S. Zhang, B. Wang, K. Davey, J. Mao, and Z. Guo, “Organic pH Buffer for Dendrite-Free and Shuttle-Free Zn-I_2_ Batteries,” *Angewandte Chemie International Edition*, 62 (2023): e202303011, https://doi.org/10.1002/anie.202303011.

16.	L. Ma, G. Zhu, Z. Wang, A. Zhu, K. Wu, B. Peng, J. Xu, D. Wang, and Z. Jin, “Long-Lasting Zinc-Iodine Batteries with Ultrahigh Areal Capacity and Boosted Rate Capability Enabled by Nickel Single-Atom Electrocatalysts,” *Nano Letters*, 23 (2023): 5272–5280, https://doi.org/10.1021/acs.nanolett.3c01310.

17.	S. J. Zhang, J. Hao, H. Li, P. F. Zhang, Z. W. Yin, Y. Y. Li, B. Zhang, Z. Lin, and S. Z. Qiao, “Polyiodide Confinement by Starch Enables Shuttle-Free Zn-Iodine Batteries,” *Advanced Materials*, 34 (2022): 2201716, https://doi.org/10.1002/adma.202201716.

18.	Y. Zhao, Y. Wang, W. Xue, R. Cheng, X. Zheng, G. Zhu, D. Hu, H. Huang, C. Hu, and D. Liu, “Unveiling the Role of Cationic Pyridine Sites in Covalent Triazine Framework for Boosting Zinc–Iodine Batteries Performance,” *Advanced Materials*, 36 (2024): 2403097, https://doi.org/10.1002/adma.202403097.

19.	Z. Chen, F. Wang, R. Ma, W. Jiao, D. Li, A. Du, Z. Yan, T. Yin, X. Yin, Q. Li, X. Zhang, N. Yang, Z. Zhou, Q.-H. Yang, and C. Yang, “Molecular Catalysis Enables Fast Polyiodide Conversion for Exceptionally Long-Life Zinc–Iodine Batteries,” *ACS Energy Letters*, 9 (2024): 2858–2866, https://doi.org/10.1021/acsenergylett.4c00992.

20.	Q. Ren, X. Tang, G. Sun, Y. Guo, Y. Li, X. Sui, and Z. Wang, “Synergetic structural optimizations of zinc anodes and electrolytes to enable zinc–iodine batteries with excellent low-temperature performances,” *ACS Nano*, 19 (2025): 29491–29502, https://doi.org/10.1021/acsnano.5c07752.

21.	C. Dong, Y. Yu, C. Ma, C. Zhou, J. Wang, J. Gu, J. Ji, S. Yang, Z. Liu, X. Xu, and L. Mai, “Tailoring zinc diatomic bidirectional catalysts achieving orbital coupling–hybridization for ultralong-cycling zinc–iodine batteries,” *Energy & Environmental Science*, 18 (2025): 3014–3025, https://doi.org/10.1039/D4EE05767H.

22.	H. Xu, R. Zhang, D. Luo, J. Wang, K. Huang, J. Chi, H. Dou, X. Zhang, and G. Sun, “Engineering eutectic network for regulating the stability of polyiodides towards high rate and long cycling zinc-iodine batteries,” *Energy Storage Materials*, 63 (2023): 103019, https://doi.org/10.1016/j.ensm.2023.103019.

23.	C. Song, Z. Gong, C. Bai, F. Cai, Z. Yuan, and X. Liu, “High performance Zn-I_2_ battery with acetonitrile electrolyte working at low temperature,” *Nano Research*, 15 (2022): 3170–3177, https://doi.org/10.1007/s12274-021-3884-z.

24.	M. Chen, H. Li, H. Xu, Z. Wu, J. Chen, H. Lin, A. Du, S. Umer, Z. Chen, N. Yue, Z. Yan, T. Yin, N. Yang, J. Lu, W. Wang, C. Yang, and Q.-H. Yang, “Redirecting iodine reduction pathways by decoupling adsorption energies for long-life Zn–I_2_ batteries,” *Energy & Environmental Science*, 19 (2026): 896–905, https://doi.org/10.1039/D5EE06963G.

25.	L. Zhang, K. Luo, J. Gong, Y. Zhou, H. Guo, Y. Yu, G. He, J.-F. Gohy, I. P. Parkin, J. Hofkens, Q. He, T. Liu, K. Müllen, and F. Lai, “Unlocking durable and sustainable zinc‐iodine batteries via molecularly engineered polyiodide reservoirs,” *Angewandte Chemie International Edition* (2025): e202506822, https://doi.org/10.1002/anie.202506822.

26.	Y. Wang, Y. Cui, M. Zhao, J. Wang, X. Liu, Y. Zhu, and Y. Yang, “Zwitterion-mediated interface chemistry for practical Zn-iodine batteries,” *Nature Communications*, 16 (2025): 5565, https://doi.org/10.1038/s41467-025-60488-9.

27.	Z. Hu, X. Wang, W. Du, Z. Zhang, Y. Tang, M. Ye, Y. Zhang, X. Liu, Z. Wen, and C. C. Li, “Crowding Effect-Induced Zinc-Enriched/Water-Lean Polymer Interfacial Layer Toward Practical Zn-Iodine Batteries,” *ACS Nano*, 17 (2023): 23207–23219, https://doi.org/10.1021/acsnano.3c10081.

28.	L. Pei, D. Xu, Y. Luo, S. Guo, D. Liu, S. Jiang, W. Zhang, and F. Cao, “Zinc single‐atom catalysts encapsulated in hierarchical porous bio‐carbon synergistically enhances fast iodine conversion and efficient polyiodide confinement for Zn‐I_2_ batteries,” *Advanced Materials* (2025): 2420005, https://doi.org/10.1002/adma.202420005.

29.	C. Wu, Y. Pan, Y. Jiao, and P. Wu, “α‐methyl group reinforced amphiphilic poly(ionic liquid) additive for high‐performance zinc–iodine batteries,” *Angewandte Chemie International Edition*, 64 (2025): e202423326, https://doi.org/10.1002/anie.202423326.

30.	B. Qi, Y. Chai, Y. Hu, Z. Liu, Y. Wang, K. Chen, C. Tan, X. Bai, K. Xie, H. Cheng, X. Chi, L. Huang, and L. Qu, “Confinement of polyiodides by dual‐functional tetrazine cathodes in Zn–I_2_ batteries,” *Angewandte Chemie International Edition* (2025): e202507497, https://doi.org/10.1002/anie.202507497.

31.	Z. Lin, H. Ding, X. Lin, M. Ye, Z. Wen, Y. Tang, X. Liu, Y. Zhang, and C. C. Li, “Phosphorylation regulation promotes bidirectional dynamic adaptive interface for achieving stable Zn–I_2_ batteries,” *Angewandte Chemie International Edition* (2025): e202506380, https://doi.org/10.1002/anie.202506380.

32.	S. Li, C. Sun, M. Zhang, R. Tang, M. Chen, W. Meng, J. Yang, Y. Kang, Z. Lv, J. Zhao, and Y. Yang, “An integrated tandem‐structured separator enables dual‐enhanced stable interfaces for long‐cycle‐life and high‐areal‐capacity aqueous zinc–iodine batteries,” *Angewandte Chemie International Edition* (2025): e202506849, https://doi.org/10.1002/anie.202506849.
